# *In Vitro* Activity of Geldanamycin Derivatives against *Schistosoma japonicum* and *Brugia malayi*

**DOI:** 10.1155/2010/716498

**Published:** 2010-12-29

**Authors:** David Wenkert, Bernadette Ramirez, Yuehai Shen, Michael A. Kron

**Affiliations:** ^1^The Department of Physiology, Michigan State University, East Lansing, MI 48824, USA; ^2^Department of Biochemistry, University of the Philippines, Manila, Philippines; ^3^Special Programme for Research and Training in Tropical Diseases (TDR), World Health Organization, Avenue Appia 20, 1211 Geneva 27, Switzerland; ^4^Biotechnology and Bioengineering Center, Medical College of Wisconsin, 8701 Watertown Plank Road, Milwaukee, WI, 53226, USA

## Abstract

Geldanamycin (GA) is a benzoquinone-containing ansamycin that inhibits heat shock protein 90. GA derivatives are being evaluated as anti-neoplastic agents, but their utility against parasites whose heat shock proteins (Hsps) have homology with human Hsp90 is unknown. The activities of four synthetic GA derivatives were tested *in vitro* using adult *Brugia malayi* and *Schistosoma japonicum*. Two of the derivatives, 17-*N*-allyl-17-demethoxygeldanamycin (17-AAG) and 17-*N*-(2-dimethylaminoethylamino)-17-demethoxygeldanamycin (DMAG), are currently in human clinical trials as anticancer drugs. Using concentrations considered safe peak plasma concentrations for these two derivatives, all four derivatives were active against both parasites. The less toxic derivative 17-AAG was as effective as GA in killing *S. japonicum*, and both DMAG and 5′-bromogeldanoxazinone were more active than 17-AAG against *B. malayi*. This work supports continued evaluation of ansamycin derivatives as broad spectrum antiparasitic agents.

## 1. Introduction

**Figure 1:**
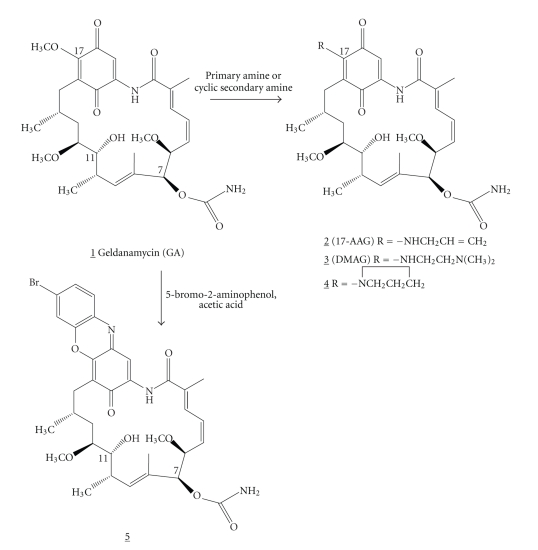
Geldanamycin (GA) chemical structure and structures and synthesis of four derivatives.

Heat shock proteins (Hsps) play critical roles in diverse biological processes including cellular development and homeostasis. Heat shock protein 90 (Hsp90) is an abundant and important eukaryotic cytosolic ATP-binding protein that serves as a chaperone in cellular processes including apoptosis and proliferation [1, 2]. Geldanamycin (GA; 1 in Figure 1) is a naturally occurring benzoquinone ansamycin, originally isolated from *Streptomyces hygroscopicus * [3]. GA binds to the ATP-binding pocket of Hsp90, specifically inhibiting ATPase activity [4, 5] and therefore it has been evaluated for its antiproliferative effects in oncogenesis *in vitro* and *in vivo* with promising application as a novel anti-cancer therapy [6, 7].Several laboratories have reported activity of GA against the Hsp of *Plasmodium falciparum* and *Brugia pahangi*; however other nematodes with conserved Hsp such as *Caenorhabditis elegans* are not affected by GA, suggesting possible conformational heterogeneity of Hsp between species [8, 9]. A less toxic derivative of geldanamycin, 17-*N*-allyl-17-demethoxygeldanamycin [10] (17-AAG; 2 in Figure 1), is now in phase II clinical trials [11, 12] in humans with neoplastic disorders. Phase I trials with 17-AAG showed safety profiles for dosing schedules with peak plasma concentrations of ca. 10 *μ*M [13–15]. A second 17-amino-substituted geldanamycin derivative, 17-*N*-(2-dimethylaminoethylamino)-17-demethoxygeldanamycin (DMAG; 3 in Figure 1), is now undergoing phase I clinical trials [16]. However, neither of these compounds is currently widely available to test their spectrum of activity against human parasites. In the Philippines, two of the World Health Organization's top ten neglected tropical parasitic diseases, lymphatic filariasis (*B. malayi* or *W. bancrofti*) and schistosomiasis caused by *Schistosoma japonicum*, account for hundreds of thousands of patient illnesses each year. Therefore, in the course of research to discover new classes of antiparasitic compounds, we studied the *in vitro* response of adult *B. malayi* and *S. japonicum* to 17-AAG and 17-DMAG.

## 2. Material and Methods

### 2.1. Geldanamycin Derivatives 2–5 Preparation

17-*N*-Allylamino-17-demethoxygeldanamycin [17-AAG, (2)], and 17-*N*-(2-dimethylaminoethylamino)-17-demethoxygeldanamycin [17-DMAG, (3)], 17-(*N*-azetidinyl)-17-demethoxygeldanamycin (4) were made in essentially quantitative yield by room temperature reaction of geldanamycin (1) with the appropriate primary or cyclic secondary amine and 5′-bromogeldanoxazinone (5) was made in moderate yield (60% yield) by heating geldanamycin with 2-amino-5-bromophenol in the presence of acetic acid [17–20] (see Figure 1).

### 2.2. Parasite Materials

Adult B.* malayi *were obtained from the laboratory of Dr. Ray Kaplan (FR3 facility, Athens, Georgia) and maintained in 12-well flat bottomed plates containing RPMI medium with penicillin-streptomycin and 0.5% DMSO at 37°C with humidity and 5% CO_2_. Mature male and female* S. japonicum* were obtained following percutaneous infection of BALB/C mice with 40 cercariae, isolated from *S. japonicum* field-infected *Oncomelania quadrasi* in the Philippines, and subsequent sacrifice of the mice at day 35 to collect mature male and female worms by saline perfusion of the mesenteric vasculature using a fine-gauged butterfly needle attached to a 50-ml sterile syringe filled with sterile normal saline.

### 2.3. Assay of Drug Toxicity

The effect of test compounds on adult *B. malayi *was monitored at final concentrations of 0.5 *μ*M, 1 *μ*M, 5 *μ*M, 0 *μ*M, 25 *μ*M. Filarial death was assessed by determining the time required for complete nonreversible cessation of motility. Each assay well contained 3 adult worms, and assays were conducted in triplicate. Experiments in triplicate were repeated at least six times. Negative control wells contained adult parasites cultured in media alone and positive control wells additionally contained 100 *μ*M albendazole. Under these conditions, negative control parasites remained actively motile for 500 hours (~20 days), whereas albendazole killed 100% of the filaria within 384 hours (16 days). Statistically significant differences in killing times were defined as *P* values <.01 (students *t-*test). Under these conditions, nonreversible cessation of motility corresponded to parasite death as measured by the MTT uptake assay.

**Figure 2:**
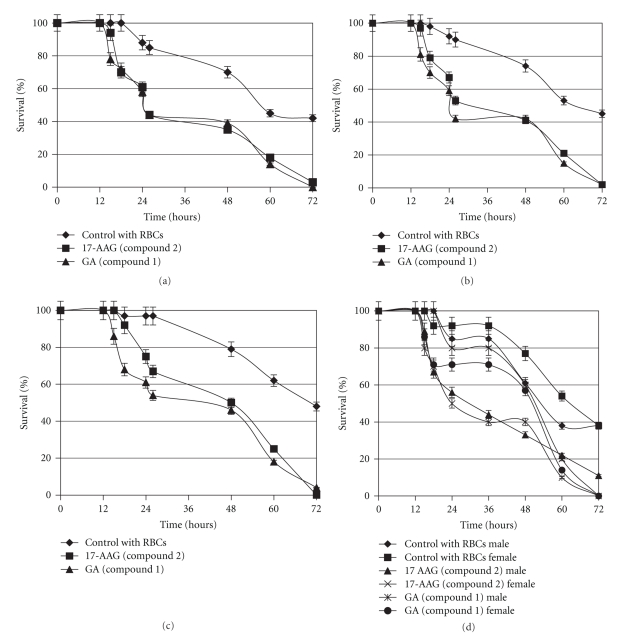
(a). Survival of paired adult male and female *Schistosoma japonicum* in the presence of human red blood cells and five micromolar geldanamycin (GA, triangles) or five micromolar 17-AAG (squares) compared to control parasites supplemented only with human red blood cells (diamonds). 50%–60% mortality was achieved at 24 hours while 90% of controls remained viable (*P* < .001). (b). Survival of individual male worms. 50%–60% mortality was achieved at 24 hours while 90% of controls remained viable (*P* < .001). (c). Survival of individual female worms. 35%–50% mortality at 24 hours while 95% of controls remained viable (*P* < .001). At 72 hours 17-AAG and GA caused 50% greater mortality versus controls. D. Survival of individual male and female worms cultured together. GA, male, star. GA female, circle. 17-AAG male, triangle. 17-AAG female, cross. At 36 hours GA caused 60% mortality in male worms while 90% of controls remained viable (*P* < .001). At 72 hours 90%–100% of GA and 17-AAG treated worms were dead versus 50% mortality in controls (*P* < .001). For (a)–(d), the data represent the mean ± S.D. of triplicate determinations in a representative experiment.

For testing of drug toxicity on *S. japonicum*, five worm pairs, five male and five female worms were placed into each well of a 24-well flat bottom plate containing 5 *μ*M of either GA or 17-AAG with 2 mL complete RPMI-1640 media supplemented with human red blood cells, 2 g/L glucose, 0.3 g/L L-glutamine, 2.0 g/L NaHCO3, 15% fetal bovine serum (heat inactivated), and 5% pen/strep (10,000 units penicillin and 10 mg/streptomycin in 0.9% NaCl). Assays were performed in triplicate and incubated in a humidified 5% CO_2_ chamber at 37°C. The physical activity of parasites (e.g., feeding behavior, movement, and viability) was recorded after 12, 15, 18, 24, 26, 48, 60, and 72 hours. Fresh culture media and test compounds were added after 18 and 36 hours. These experiments were all repeated at least five times. Experiments were repeated using paired male and female worms, as well as individual male worms and female worms. Effects of GA derivatives were highly reproducible using paired adult worms, individual female, and individual male worms (see Figure 2). Each graphic point represents three individual experiments.

## 3. Results

Both GA and 17-AAG killed adult male and female *S. japonicum* at a concentration of 5 *μ*M (Figures 2(a)–2(d)). The effects of both compounds were identical using paired worms, isolated males, isolated females, and isolated larvae (cercariae, data not shown.) The effects of both compounds were highly reproducible within 12–24 hours, even though the longevity of adult *S. japonicum* cultured in minimal media is only 40%–50% at 72 hours. Lower concentrations of each compound were not tested due to a scarcity of adult *S. japonicum*. 

**Figure 3:**
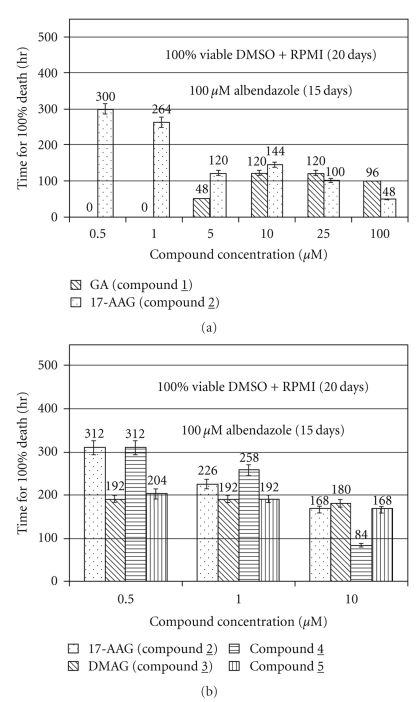
(a) Effect of Geldanamycin and 17-AAG on adult *Brugia malayi in vitro.* 17-AAG killed *B. malayi* adults faster and at concentrations two hundred times lower than albendazole (positive control). Statistically significant differences in rates of killing were observed for 5–100 *μ*M GA and 17-AAG versus 100 *μ*M albendazole (positive control, *P* < .001). 500 nM and 1000 nM GA and 17-AAG were equivalent to 100 *μ*M albendazole at 280–300 hours. (b) Effect of 17-AAG compared to three other GA derivatives on adult *B. malayi in vitro*. Negative control worms (i.e., no drug) remain viable for 500 hours and 100 *μ*M albendazole kills parasites by 15 days. All three concentrations of the four GA derivatives killed adult *B. malayi* faster than 100 *μ*M albendazole (positive control, *P* < .001). Numbers above each bar are mean hours to 100% killing. Compounds 3 (DMAG) and 5 (5′-bromogeldanoxazinone) were more active against *B. malayi* than 17-AAG at 500 nM (*P* < .001). All four compounds at each concentration tested (500 nM—10 *μ*M) killed filarial more quickly than 100 *μ*M albendazole (positive control, *P* < .001). For (a) and (b), the data represent the mean ± S.D. of triplicate determinations in a representative experiment.

Adult *B. malayi* were killed quicker by both GA and 17-AAG and at concentrations significantly lower than 100 *μ*m of albendazole (positive control) (Figure 3(a)). Macrofilaricidal concentrations of 17-AAG were 10–20 times lower than the peak plasma concentrations (10 *μ*M) that are reported as safe in phase I clinical trials. All four GA derivatives were macrofilaricidal at 500 nM, the lowest concentration tested (Figure 3(b)). DMAG and compound 4 had the most rapid macrofilaricidal effect (9 days) compared to 100 *μ*M albendazole (16 days) under these conditions (*P* < .01 for all concentrations versus albendazole). Most compounds at concentrations greater than 10 *μ*M exhibited some precipitation in RPMI with 0.5% DMSO.

## 4. Discussion

**Figure 4:**
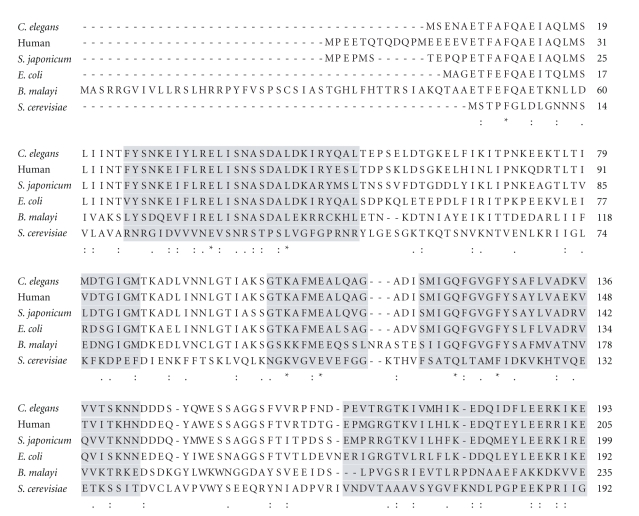
Multiple sequence alignment (Clustal W) of five eukaryotic and one prokaryotic heat shock proteins (Hsp90). Grey-shaded areas indicate residues that comprise the geldanamycin-binding pocket that can be conserved across species [4, 21]. *S. cerevesiae *: *Saccharomyces cerevisiae*; *E. coli *: *Escherichia coli*; *S. japonicum *: *Schistosoma japonicum*; *B. malayi *: *Brugia malayi*;* C. elegans *: *Caenorhabditis elegans*.

Human heat shock protein 90 (Hsp90) belongs to the “GHKL” group of ATPases [22]. The ATP-binding site of this group of proteins is uniquely affected by GA. Molecular chaperones such as Hsps play critical roles in diverse biological processes including cellular development and homeostasis. A high degree of conservation of same and similar amino acid residues is exhibited in the ATP-binding pocket in the known protein sequences of Hsp90 of various species (Figure 4). The antiparasitic effect of GA derivatives suggests that similar life-dependent ATP binding sites are being affected in such organisms. 

The importance of Hsps in the biology of human and veterinary parasites has been reported previously in numerous genera including *Leishmania, Trypanosoma, Plasmodium, Schistosoma,* and various nematodes. Devaney et al., reported macrofilaricidal activity of GA against the dog and cat filaria, *Brugia pahangi,* at nanomolar levels [23]. Also, it was noted that while GA clearly bound to *B. pahangi* Hsp, the free living nematode *Caenorhabiditis elegans* did not bind GA despite a high degree of conservation between the nematode Hsp sequences [8]. A recent study of the geldanamycin-binding ability of Hsp90, derived from a number of nematodes, found that in contrast to some obligate parasites, the free-living species and the parasitic species having free-living environmental larval stages that were tested did not bind geldanamycin [9]. In the case of *P. falciparum*, a systems analysis of chaperone networks combining experimental interactome, *in silico* and yeast two hybrid assays, facilitated predictions and functional assignment for Hsp70-Hsp40 interactions and the Hsp90 and Hsp100 families [24]. In *P. falciparum*, GA is known to inhibit all intraerythrocytic stages and kills the parasites within a single developmental cycle [25, 26].

The parasite killing assays employed in these studies differ slightly from those used by other WHO collaborating groups. For example, filaria killing studies are sometimes conducted in the presence of monkey kidney cell feeder layers up to a maximum of 120 hours, to control for the toxicity of test compounds [27]. However, in the case of most GA derivatives tested herein, (1) the lack of toxicity to human cells was already established, (2) the removal of serum from culture media permits longer *in vitro* culture as noted, up to 500 hours, and (3) such serum removal allows the evaluation of lipophilic compounds which can bind to albumin or other serum proteins and can obscure their inherent activity against filaria [28]. Recent advances in medicinal chemistry include strategies to make use of lipophilic drugs [29] which by themselves could have lower peak plasma concentrations and greater volumes of distribution.

In the case of schistosomes, WHO collaborating centers generally use a 5-day assay without red blood cells in the culture media when evaluating the activity of lead compounds against *S. mansoni* and *S. hematobium* [30]. However, *S. japonicum* adult worms are much more difficult to maintain *in vitro *using the same *in vitro* conditions used for *S. mansoni*. Cercariae, a different life cycle stage of *S. japonicum*, are similarly affected by GA derivatives as well (data not shown).

Antiparasite drug discovery strategies commonly focus on identification of molecular targets unique to the parasite in the belief that host toxicity or cross-inhibition of host pathways will be minimized. While such approaches may ultimately be effective, an alternative approach is to target proteins or pathways that are common to both parasite and host, but which may have evolved over time features, domains, or alternative functions that are unique to the species. When such a molecule is acknowledged as a rationale target for drug discovery in more than one disease, this approach has the benefit of a broader base of scientific and medicinal chemistry infrastructure on which to develop lead antiparasite compounds. Additionally, the ubiquity of such target proteins among parasites allows parallel development of therapeutics that can be effective in the treatment of differing parasitic infections. Thus the next steps in the evaluation of GA derivatives as parasitic agents must include pharmacokinetic and *in vivo* killing experiments to determine effective *in vivo* dosing regimens using the *S. japonicum* mouse model and either the *B. malayi* transplanted jird model or the L3-induced *B. malayi* infection in *Mastomys coucha* [31]. Additionally, to be determined are the degree to which the antiparasitic effect of GA and its derivatives is dependent on penetration to target, binding affinity, unique heat shock protein dependent cochaperones, or possible additional GA targets. Although the antifilarial activity of various anticancer compounds obtained from the U.S. National Cancer Institute has been previously studied, neither GA or its derivatives were so studied [32]. An alternative strategy for activity evaluation of compounds against *S. japonicum* would include a side-to-side comparison with *S. mansoni*, which is easier to maintain *in vitro *and can demonstrate more easily the *in vitro* effects of a positive control drug such as praziquantel [33]. (*S. mansoni*, however, does not exist in the Philippines.)

A fluorescence polarization assay for Hsp90 activity was recently reported by Taldone et al. [34]. Whole worm extracts of *Brugia pahangi* were used in a modified assay previously validated for discovery of antitumor Hsp90 inhibitors, and this assay was validated using soluble *B. pahangi* extracts. Though reported suitable for high-throughput screening, compounds identified by this *in vitro* method do not take into account chemical properties that would facilitate or inhibit transport of antiparasite compounds across the cuticle of adult worms. Also, soluble extracts of the human parasite, *B. malayi*, were not systematically compared to the extracts from *B. pahangi*, an animal parasite. 

Although compounds 4 and 5 have not been tested in humans nor are their pharmacokinetic parameters yet known, compounds 17-AAG (2) and DMAG (3) have the advantage of having been tested in humans and were found to be highly active against human tumor cells *in vitro* and accordingly were and are being evaluated in human clinical trials of cancer chemotherapy. The obtained human safety and tolerance data of the latter two drugs at concentrations found lethal to S. *japonicum* and B. malayi gives added reason for investigation of these and other Hsp90 inhibitors as antiparasitic therapeutic agents. Additionally, the recent finding of the reduced and protonated hydroquinone version of 17-AAG having water solubility and equivalent efficacy as a Hsp90 inhibitor allows entré to similar water soluble analogs of other 17-*N*-alkylamino-17-demethoxygeldanamycin derivatives [35]. Modulation of heat shock proteins is increasingly being recognized as having various demonstrated and potential beneficial therapeutic effects [36]. Our *in vitro* studies support the activity of GA derivatives against the Hsp of two important new groups of human parasites.
